# Identifying Potential New Gene Expression-Based Biomarkers in the Peripheral Blood Mononuclear Cells of Hepatitis B-Related Hepatocellular Carcinoma

**DOI:** 10.1155/2022/9541600

**Published:** 2022-02-28

**Authors:** Vahdat Poortahmasebi, Ahmad Nejati, Mohammad Foad Abazari, Mohsen Nasiri Toosi, Azam Ghaziasadi, Nader Mohammadzadeh, Ahmad Tavakoli, Azam Khamseh, Navid Momenifar, Omid Gholizadeh, Mehdi Norouzi, Seyed Mohammad Jazayeri

**Affiliations:** ^1^Infectious and Tropical Diseases Research Center, Tabriz University of Medical Sciences, Tabriz, Iran; ^2^Department of Bacteriology and Virology, School of Medicine, Tabriz University of Medical Sciences, Tabriz, Iran; ^3^Research Center for Clinical Virology, Tehran University of Medical Sciences, Tehran, Iran; ^4^Department of Virology, School Public Health, Tehran University of Medical Sciences, Tehran, Iran; ^5^Liver Transplantation Research Center, Tehran University of Medical Sciences, Tehran, Iran; ^6^Central Laboratory of East Azerbaijan Province, Tabriz University of Medical Sciences, Tabriz, Iran; ^7^Research Center of Pediatric Infectious Diseases, Institute of Immunology and Infectious Diseases, Iran University of Medical Sciences, Tehran, Iran; ^8^Human and Animal Cell Bank, Iranian Biological Resource Center (IBRC), ACECR, Tehran, Iran

## Abstract

**Objective:**

The analysis of the gene expression of peripheral blood mononuclear cells (PBMCs) is important to clarify the pathogenesis of hepatocellular carcinoma (HCC) and the detection of suitable biomarkers. The purpose of this investigation was to use RNA-sequencing to screen the appropriate differentially expressed genes (DEGs) in the PBMCs for the HCC.

**Methods:**

The comprehensive transcriptome of extracted RNA of PBMC (*n* = 20) from patients with chronic hepatitis B (CHB), liver cirrhosis, and early stage of HCC (5 samples per group) was carried out using RNA-sequencing. All raw RNA-sequencing data analyses were performed using conventional RNA-sequencing analysis tools. Next, gene ontology (GO) analyses were carried out to elucidate the biological processes of DEGs. Finally, relative transcript abundance of selected DEGs was verified using qRT-PCR on additional validation groups.

**Results:**

Specifically, 13, 1262, and 1450 DEGs were identified for CHB, liver cirrhosis, and HCC, when compared with the healthy controls. GO enrichment analysis indicated that HCC is closely related to the immune response. Seven DEGs (TYMP, TYROBP, CD14, TGFBI, LILRA2, GNLY, and GZMB) were common to HCC, cirrhosis, and CHB when compared to healthy controls. The data revealed that the expressions of these 7 DEGs were consistent with those from the RNA-sequencing results. Also, the expressions of 7 representative genes that had higher sensitivity were obtained by receiver operating characteristic analysis, which indicated their important diagnostic accuracy for HBV-HCC.

**Conclusion:**

This study provides us with new horizons into the biological process and potential prospective clinical diagnosis and prognosis of HCC in the near future.

## 1. Introduction

Hepatitis B virus (HBV) infection is the leading cause of liver cirrhosis and hepatocellular carcinoma (HCC) worldwide with an annual mortality rate of 1 million deaths. Despite being successful for more than three decades in preventing hepatitis B infection, HBV is estimated to affect about 240 million people worldwide. Approximately 50% of patients with chronic hepatitis B infection (CHB) will progress to liver cirrhosis and the annual incidence rate of HCC in this group is 4% [1]. HCC is one of the most frequent cancers and the fourth leading cause of cancer-related death worldwide [2]. The majority of patients with HCC are diagnosed with advanced stage, restricting treatment choices. Therefore, the identification of new biomarkers that improve the diagnostic performance of HCC is crucially required.

Chronic hepatitis is a dynamic procedure indicating the interaction between HBV life cycle and the host genetic/immune response, and not all patients with HBV infection have severe disease. Investigations are analyzing viral or patient genetics gene expression data in relation to disease prognosis and prediction. The tumor microenvironment and its alterations are critical for cancer progression and associated with an aggressive phenotype of several tumor types. The cancer tissue is recognized as a complex cellular community that includes a variety of cells, including the surrounding blood vessels, immune cells, fibroblasts, signaling molecules, and the extracellular matrix, which is referred to as the tumor microenvironment. Therefore, these genomic and biological variations within a tumor lesion are referred to as intratumor heterogeneity. In contrast, intertumor heterogeneity refers to tumors from different patients whose altered genotype and phenotype are induced by diverse etiological and environmental factors [3]. It has been indicated that HCC cell populations represent greater genomic heterogeneity. Intratumoral heterogeneity of HCC, which can directly interact with tumor cells and affect the therapeutic response, has been considered as a critical aspect of cancer [4].

To shed light on these inconclusive problems of HCC tumor heterogeneity, instead of the tumor tissue, the peripheral blood mononuclear cells (PBMCs) can be employed as an alternate tissue to throw more light on these contradictory issues, minimize information misinterpretation, and produce promising outcomes. PBMCs might be effective for boosting the reliability of the results by lowering intratumor heterogeneity due to their simple makeup. The establishment of next-generation RNA-sequencing (RNA-seq) technology has improved our understanding of the role and the molecular mechanism of gene biomarkers in human cancer and has been broadly performed for cancer research and detection of novel biomarkers for cancer diagnosis and prognosis [5, 6]. Nowadays, there is a continued need for the discovery of specific blood biomarkers to assist in the minimally invasive detection of cancer and the monitoring of the effectiveness of cancer therapy [7, 8]. Undoubtedly, the availability of PBMC samples to analyzing of molecular biology will pave the way for personalized medicine [9]. The use of blood as a surrogate tissue, which can be obtained with a noninvasive method, is a marvelous alternative to liver biopsies. Several investigations have demonstrated that the expression levels of specific mRNA in PBMCs can serve as a biomarker of several diseases, including Parkinson's disease [10], cancer [11], leukemia [12], and heart failure [13].

In the current study, we hypothesized that a dysregulation in the transcriptome profile of PBMCs occurs in patients with different phases of HBV diseases, and identification of HCC-specific gene subsets in PBMCs could be potentially useful in the early detection of this cancer. Subsequently, additional specimens from another 75 hepatitis B patients and 25 healthy subjects were also enrolled for verification by quantitative real-time PCR (qRT-PCR).

## 2. Methods

### 2.1. Patients

In the present investigation, RNA-seq was performed to evaluate the gene expression of PMBCs from healthy subjects (*n* = 5), patients with CHB (*n* = 5), cirrhosis (*n* = 5), and early stages of HCC (*n* = 5). Another 100 subjects (25 per group) were assigned for validation purposes. All samples in the present study were obtained from Imam Khomeini Hospital Complex of Tehran University of Medical Sciences (Tehran, Iran). Patients with a history of hepatic steatosis, diabetes, and other metabolic disorders were excluded from the study. All patients had no evidence of coinfection with hepatitis C and D virus and human immunodeficiency virus (HIV). Also, patients with other liver diseases, pregnancy, Wilson disease, previous liver transplantation, autoimmune liver diseases, and alcoholic liver disease were excluded from the study. Before the time of specimen collection, all patients were signed an informed consent provided by the Local Ethics Committee. The study was evaluated and approved by the Ethical Committee of National Institute for Medical Research Development (NIMAD) [ID number: IR.NIMAD.REC.1396.396].

### 2.2. Serological Assessments and Liver Function Test

Viral hepatitis markers were measured by enzyme-linked immunosorbent assay (ELISA) including HBsAg and anti-HBc, anti-HCV, and anti-HDV (Dia.Pro, Milan, Italy) according to manufacture protocol. All samples were retested by another ELISA kit (Acone, San Diego, CA, USA). To examine the serum levels of alanine aminotransferase (ALT) and aspartate aminotransferase (AST), commercial kits were used.

### 2.3. HBV DNA Quantification, Sequencing, and Genotyping of HBV

HBV DNA was extracted from a 200 *μ*l aliquot of serum using a high pure viral nucleic acid kit (Roche, Germany) according to its commercial protocols. For all subjects, a quantitative TaqMan RT-PCR was applied using fast-track diagnostics kits (FTD, Luxembourg) according to the manufacturer's recommendations. Afterward, HBV surface gene standard nested-PCR was carried out on samples using specific primers as previously described [14]. The nucleotide sequences of the HBsAg encoding component were determined bilaterally by the 3130 Genetic Analyzer (Genetic Analyzer ABI- 3130 DNA Sequencer, Foster City, CA, USA). In order to study HBV genotyping, strain sequence accessed from the NCBI site was used as a reference (Accession number: GQ183486). The phylogenetic tree was created utilizing MEGA X software.

### 2.4. RNA-Sequencing, Library Preparation, Next-Generation RNA-Sequencing, and Bioinformatics Analysis

The 4 mL fresh EDTA-blood samples were obtained from all subjects before use in the study procedures. PBMCs were isolated using Ficoll density gradient centrifugation (Sigma-Aldrich, Germany) according to the manufacturer's instructions. Total RNA was extracted using the TRIZOL reagent (Ambion, USA) according to the protocol provided by the manufacturer. Total RNA concentrations were quantified spectrophotometrically at 260/280 nm using a NanoDrop spectrophotometer (Thermo Fisher Scientific, Waltham, MA, USA). The quality of the isolated RNA was assessed by electrophoresis on a 1% agarose gel containing a safe stain, and RNA integrity was evaluated using the Bioanalyzer 2100 (Agilent, Santa Clara, CA, USA). All 20 RNA samples were sent to the Novogene Company in Hong Kong, for the purpose of performing library construction and next-generation RNA-seq facility. The cDNA libraries were paired-end (PE) sequenced (2 × 150) using an Illumina HiSeq 4000.

All raw RNA-seq data analyses were performed using conventional RNA-seq analysis tools. The quality of the FastQ files was assessed with FastQC version 0.11, and all Phred quality scores were more than 20 (base call accuracy: 99%). Cleaning and trimming of low-quality reads were performed by Trimmomatic version 0.36. All RNA-seq clean files were aligned onto the human reference genome (version hg38) using HISAT2 (version 2.2.1.0). Gene read counts were performed by HTSeq version 0.9.1. Analysis of differentially expressed genes (DEGs) was carried out using R software and DESeq2 package Bioconductor [15]. We normalize the gene expression data with Log_2_ fold change (FC > 1) along with adjusted *P* value (false discovery rate, [FDR] ≤ 0.05) and also represent the annotation information.

### 2.5. Gene Enrichment Analysis and Protein-Protein Interaction Networks

Gene ontology (GO) studies were performed utilizing Database for Annotation, Visualization, and Integrated Discovery (DAVID) (https://david.ncifcrf.gov/summary.jsp) [16]. A protein-protein interaction (PPI) network was generated for each normal-CHB, normal-cirrhosis, normal-HCC, CHB-cirrhosis, and cirrhosis-HCC states using the BisoGenet plugin of Cytoscape software (Version 3.9.0) [17]. Topological features of each PPI were measured using NetworkAnalyzer, a network analysis plugin of Cytoscape, to detect critical functional hub genes within the networks [18]. We performed important measures including Degree Centrality, Betweenness Centrality, and Closeness Centrality [19].

### 2.6. Validation by qRT-PCR

The genes identified based on the primary high-throughput sequencing data were evaluated in additional 100 participants (25 per group) using qRT-PCR. Total RNA was isolated from all samples as described before. Contaminating genomic DNA was removed with a DNase I treatment (Qiagen, Germany). RNA was reverse transcribed to cDNA using QuantiTect Reverse Transcription Kit (Qiagen, Germany) according to the manufacturer's protocols. The qRT-PCR was carried out on the RotorGene 6000 device (Corbett, Mortlake, New South Wales, Australia), and qRT-PCR reactions were performed with RealQ Plus 2x Master Mix Green (Ampliqon, Denmark) using specific gene primers (Metabion, Germany). Primer pairs used in this study are listed in Table 1. PCR program was as follows: predenaturation at 95°C for 10 min, followed by 40 cycles of PCR followed by 40 cycles of 95°C for 15 s, 57°C for 30 s, and 68°C for 30 s. Glyceraldehyde-3-Phosphate Dehydrogenase (GAPDH) was used as a housekeeping gene for standardizing targeted mRNA expression. mRNA expression patterns were analyzed according to the 2^−ΔΔCT^ method [20]. The relative values of the gene of interest were represented as fold change (FC) to compare mRNA levels between patients.

### 2.7. Statistical Analysis

GraphPad Prism software, version 5 (GraphPad software, Inc, La Jolla, California), was used for both plotting graphs and statistical analysis. Continuous variables were expressed as the mean ± standard deviation (SD) or median and were compared using one-way ANOVA analysis or independent *t*-test. Genes were clustered using principal component analysis (PCA) by R software. PCA evaluates the similarity of the gene expression profiles of PBMCs from different subjects. Subsequently, common expression of DEGs between different states and Venn diagrams was drawn using the online tools available through the VENNY 2.1 (https://bioinfogp.cnb.csic.es/tools/venny/index.html). To check the significance of all comparisons, a *P* value <0.05 was considered statistically significant. Moreover, the area under the receiver operating characteristic (ROC) curves was calculated using statistical analysis IBM SPSS Statistics 24 (SPSS Inc., Chicago, Illinois, USA) to evaluate the diagnostic accuracy of the selected DEGs analyzed.

## 3. Results

### 3.1. General Characteristics of Patients

We performed RNA-seq to display the global gene expression profiles of 5 PBMC samples of patients with CHB, 5 PBMC samples of patients with liver cirrhosis, 5 PBMC samples of patients with HCC, and 5 PBMC samples of healthy subjects. The mean age for total participants was 48.85 ± 11.88. The difference between the genders and ages of subjects was not significant (*P* value > 0.05; Table 2). In terms of HBV viral loads, significant associations were found between patients in terms of HBV viral loads (*P* value <0.001). Among cirrhosis and HCC patients, 3 (60%) and 3 (60%) were HBeAg positive, respectively. In terms of liver injuries markers, we found statistical associations between ALT and AST between groups (*P* value <0.001).

**Figure 1:**
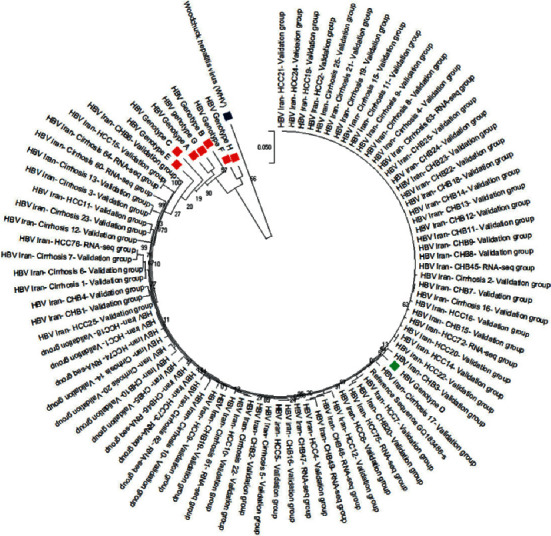
Phylogenetic tree. Maximum Likelihood phylogenetic tree analyses of HBsAg using the Kimura two-parameter substitution test with 1,000 bootstrap sampling in the MEGA X software. Genotyping was performed by phylogenetic analysis with reference sequences of HBV genotypes (a–h). All of the HBV reference sequences are represented. Woodchuck hepatitis virus (WHV) was used as an outgroup.

Subsequently, 100 individuals were considered for validation purposes (25 subjects per group). The demographic and clinical features of the validation group are summarized in Table 3. The levels of HBV DNA, ALT, and AST were not significantly different between the sequencing and validation groups ( ± -value >0.05; result not shown). Direct sequencing of 681-bp HBsAg indicated that the HBV genotype was D in all of the HBV patients (Figure 1). However, nine samples did not have sufficient material for further sequence analysis.

### 3.2. Detection of DEGs in HCC Patients Compared to CHB, Cirrhosis, and Healthy Controls

To determine alterations in the gene expression profile associated with hepatitis B diseases, the transcriptomes of PMBCs from HCC patients, cirrhosis patients, CHB patients, and healthy subjects were carried out using high-throughput sequencing. RNA-seq was obtained from 19,430,539 to 29,921,566 raw paired-end reads that were aligned to the human reference hg38, indicating 18,001,907 to 28,031,232 uniquely mapped reads. Detailed information regarding each sample was also recorded (Table 4). After processing of noninformative data, 13 DEGs including 12 upregulated genes and 1 downregulated gene were detected in CHB groups compared to healthy controls. 1262 DEGs in cirrhosis (933 genes upregulated and 329 genes downregulated) and 1450 DEGs in HCC (1010 genes upregulated and 440 genes downregulated) were identified, relative to healthy controls. The number of detected DEGs was increased with the progression of HBV disease. Also, 408 transcripts were differentially expressed between cirrhosis and CHB patients, where 344 genes were upregulated and 64 genes were downregulated. Finally, 133 DEGs including 74 upregulated genes and 59 downregulated genes were detected in HCC patients compared to cirrhosis patients.

**Figure 2:**
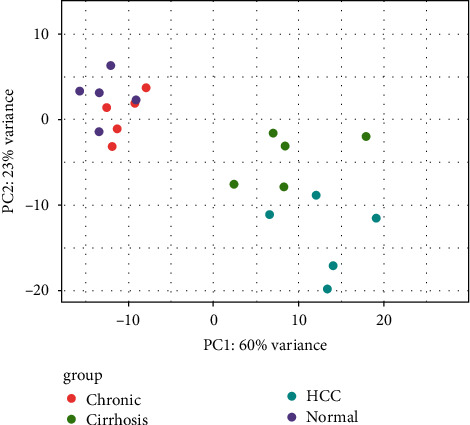
Plotting PCA. PC1 and PC2 plots indicate the characteristics of data such as nonlinearity and departure from normality. PC1 and PC2 are examined for each sample and plotted. In PC1, all of the samples (normal, CHB, cirrhosis, and HCC) were gathered, and each of them had similar effects, demonstrating their similarity. PC1 demonstrated the general features of the expression profile of HCC. Moreover, there are certain differences in gene expression levels between the groups of PC2 with respect to HCC samples and non-HCC samples.

PCA clustering analysis was used to evaluate the features of gene expression levels of dysregulated genes. PCA reduced dimensionality creating a few linear combinations of all data that are named ‘principle components'. In the present study, PCA analysis distinguished samples into several clusters. However, CHB samples were located in the healthy control cluster, representing an overlap between the global expression profiles of healthy control and CHB inactive carriers. As revealed in Figure 2, the first principal component (PC1) accounted for 60% of the total variance of the data, and the other principal component (PC2) accounted for 23%.

### 3.3. Gene Enrichment Analysis of HCC Associated DEGs

**Figure 3:**
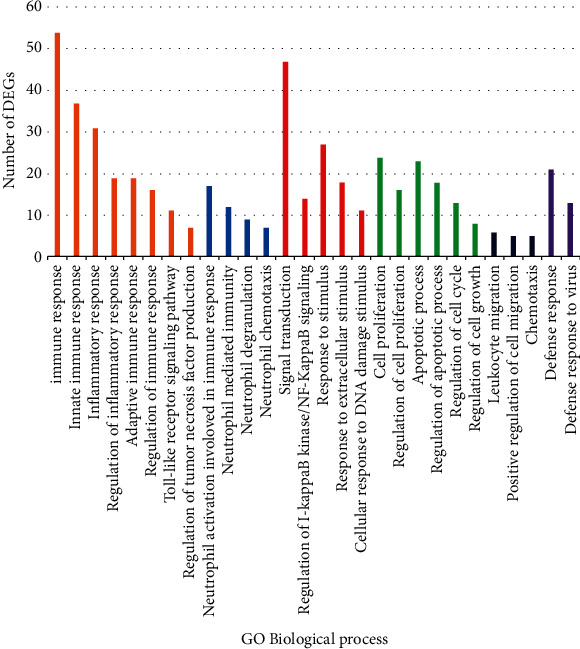
Gene ontology enrichment analyses of the DEGs which were involved in the HCC.

Gene ontology (GO) analysis was carried out with the DAVID Functional Annotation Tool to identify the significant pathobiological processes which were implicated in the HCC. The purpose of the DAVID online system is to identify the causal biological processes in a given list of input human genes. As seen in Figure 3, we found that HCC is statistically associated with several pathologic processes, including immune response, neutrophil-mediated immunity, signal transduction, cell proliferation, leukocyte migration, and defense response.

**Figure 4:**
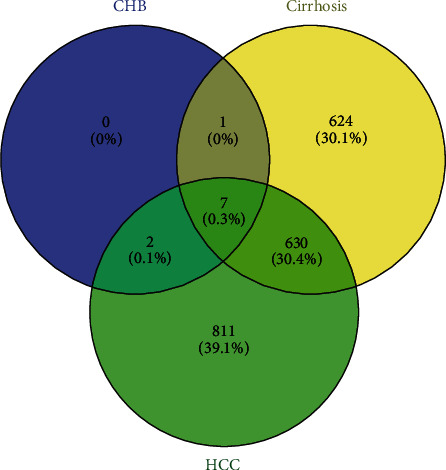
Venn-Diagram analysis to identify the common DGEs. 1 DEG (HLA -H) was common to cirrhosis and HCC, which was upregulated in these two groups, and 7 DEGs (TYMP, TYROBP, CD14, TGFBI, LILRA2, GNLY, and GZMB) were common to CHB, cirrhosis, and HCC, where only 1 DEG was downregulated (CD14).

Furthermore, we draw a Venn-Diagram for the detection of the common DGEs. As seen in Figure 4, when compared to healthy controls, only 7 DEGs, including 6 up-DEGs (TYMP, TYROBP, TGFBI, LILRA2, GNLY, and GZMB) and 1 down-DEG (CD14) were common to CHB, liver cirrhosis, and HCC. Expectedly, we found that these genes were involved in the immune response, which was shared by CHB, cirrhosis, and HCC, demonstrating that these 7 genes had potential values to diagnose HCC. Thereafter, qRT-PCR was used for extra verification of these 7 genes.

### 3.4. Topological Analysis of DEGs

By integrating the regulatory relationships obtained from the BisoGenet plugin of Cytoscape software, PPIs were assayed for each DEG list obtained from processing normal-CHB, CHB-cirrhosis, and cirrhosis-HCC gene expression profiles. However, we did not find significant PPI in normal-CHB (DEGs = 13) and cirrhosis-HCC (DEGs = 133) states. For PPI of CHB-cirrhosis, the Cytoscape was delivered a network with 408 nodes (genes) and 149 edges (interactions). Moreover, for PPI of normal-cirrhosis, CHB-cirrhosis, normal-HCC, and CHB-HCC, BisoGenet has yielded a network, which is indicated in Table 5.

After determination of each PPI's subnetwork, the most integrated subnetworks were observed in PPI of CHB-cirrhosis, normal-cirrhosis, normal-HCC, and CHB-HCC. All generated PPIs were topologically evaluated using the NetworkAnalyzer plugin of the Cytoscape.  Table 6 listed the top hub DEGs of each PPI determined by credible topological parameters. Some of the important hub genes in the BisoGenet-derived PPI for liver cirrhosis and HCC diseases were E2F1, TAL1, CEBPB, ELF1, RAD21, CEBPB, and MYC. Functional analysis of the hub genes indicated that these genes were mainly involved in immune response and cell proliferation. Several genes including E2F1, ELF1, and USF1 with high degrees of centrality were common hub genes in the majority of networks.

### 3.5. Validation of the mRNA Expression Levels by qRT-PCR

**Figure 5:**
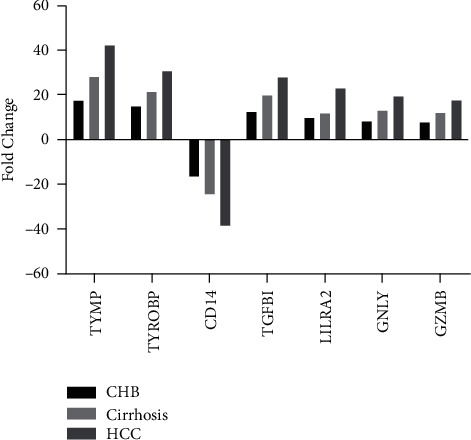
The expression level comparison of selected DEGs for validation.

To validate the RNA-seq results, qRT-PCR was performed to verify the expression levels of DEGs in PBMCs of other CHB patients (*n* = 25), liver cirrhosis patients (*n* = 25), HCC patients (*n* = 25), and a control subject (*n* = 25). Ten common DEGs (TYMP, TYROBP, CD14, TGFBI, LILRA2, GNLY, and GZMB) obtained from Venn-Diagram analysis were assigned to an evaluation in the validation cohort. The RNA-seq results revealed that 6 out of 7 common DEGs were significantly upregulated in PBMCs of all HBV patient groups when compared to healthy subjects, as indicated in Table 7. Only 1 DEG (CD14) was downregulated in these three groups. The qRT-PCR data indicated similar changes (Figure 5), and statistical differences were found between the patient's groups and the healthy controls in all seven genes. Specifically, the relative expression changes of six DEGs were increased >10-fold in HCC patients compared with cirrhosis and CHB patients.

**Figure 6:**
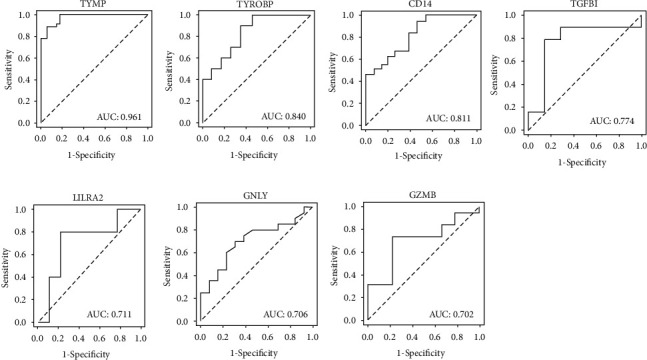
ROC curves for the seven DEGs: TYMP, TYROBP, CD14, TGFBI, LILRA2, GNLY, and GZMB.

To evaluate the potential value of significantly and differentially expressed common genes for HCC diagnosis, the qRT-PCR results from 75 cohort patients with HCC, cirrhosis, and CHB were then subjected to receiver operating characteristic analysis, which revealed that these genes have credibly predictive power. As indicated in Figure 6, the areas under the ROCs (AUROCs) were 0.961 (95% CI 0.931–0.991 *P* value < 0.001) for TYMP, 0.840 (95% CI 0.770–0.910 *P* value < 0.001) for TYROBP, 0.811 (95% CI 0.766–0.856 *P* value < 0.001) for CD14, 0.774 (95% CI 0.694–0.854 *P* value < 0.001) for TGFBI, 0.711 (95% CI 0.621–0.801 *P* value < 0.001) for LILRA2, 0.706 (95% CI 0.630–0.782, *P* value < 0.001) for GNLY, and 0.702 (95% CI 0.625–0.779, *P* value < 0.001) for GZMB.

## 4. Discussion

Human hepatocellular carcinoma is one of the most common cancers worldwide and the fourth most frequent cause of cancer-related mortality [2, 21]. The HCC is usually diagnosed at advanced stages and has a poor prognosis. Thus, seeking specific and reliable biomarkers for early diagnosis of HCC is essential to identify patients who need early treatment. There are many hosts and virological factors such as high viral load, gender, and HBsAg levels that play a critical role in the occurrence and development of HCC. HBV viral load evaluations and genotyping have become important for predicting HBV disease severity, performed for treatment guidelines detecting the emergence of antiviral drug resistance [22, 23]. In our study, direct sequencing results from HBsAg sequences indicated that all belonged to HBV genotype D. Recent investigations from several regions of Iran revealed that genotype D is the only circulated HBV genotype in the country [24]. The present study used the easily available and noninvasive PBMC specimens to evaluate the RNA-seq transcriptome profiles of HBV-derived chronic hepatitis, liver cirrhosis, and HCC. Although several researchers investigated the microarray and RNA-seq transcriptomes of PBMCs from HCC patients [25–29], to our knowledge, comparative RNA-seq transcriptome analysis of PBMCs in different stages of HBV infection has not been reported.

In the present study, gene expressions of PBMCs from all 20 subjects resulted from RNA-seq and were then analyzed using computational tools. Afterward, selected genes were verified by qRT-PCR in another validation cohort including 25 CHB, 25 cirrhosis, 25 HCC patients, and 25 healthy subjects. Finally, to investigate the expression levels of validated genes as diagnostic or prognostic biomarkers of HCC, we performed an ROC curve assessment. After RNA-seq analysis, we examined dysregulated genes in PBMCs from HBV patients. When compared with the normal subjects, 13, 1262, and 1450 DEGs were identified for CHB, liver cirrhosis, and HCC (Table 1), demonstrating an increasing number of DEGs with the development of HBV diseases. The increased number of DEGs with the progression of HBV infection is probably affected by the growth of circulating tumor cells (CTCs), and the number of DEGs is probably able to be utilized for supervising the development of HCC [30]. To clarify the different clusters of HCC associated DEGs, functional enrichment analysis was performed. The biological processes such as immune response, neutrophil-mediated immunity, signal transduction, cell proliferation, leukocyte migration, and defense response are also statistically activated in HCC and listed at the top when ranked by the DAVID annotation tool. Inconsistent with our study, other researchers reported that HCC is related to immunity, and the majority of immune functions act may be essential roles in the occurrence and progression of HCC. Recently, Shen et al. reported that two biological processes in the PBMCs including immune system and response to stimulus were significantly implicated in the hepatic carcinoma with metastasis [26]. In addition to the pathological processes obtained from gene ontology, we integrated RNA-seq data with high-throughput PPI data to identify deregulated networks in the HCC. The analysis of the molecular network revealed that several DEGs are critical hub genes implicated in immune responses. These findings suggested that HCC is closely linked to immune responses. The mechanisms behind these alterations are poorly understood. A recent study reveals that there is a direct relationship between cancer cells and peripheral immune cells [31]. The cytokines should be implicated in the connection between cancer cells and PBMCs [32]. Recent researches indicate that the malignant cells are responsible for the alterations in the secretion of the cytokines from PBMCs and might be associated with cancer progression [33]. This suggests a dynamic relationship between cell-to-cell interaction and differences in PBMCs gene expressions.

RNA-Seq is an evolving novel technology that makes the employment of next-generation sequencing to deal with gene expression profiling. Gene expression analysis is a powerful tool that is used for a better understanding of the disease progression in a host. Also, it is a robust and effective method for genome study and major functional gene and molecular markers' identification [34]. The success of the treatment of HCC patients goes through the best understanding of the HCC pathogenesis. In the new horizon of genomic oncology, genetic biomarkers are becoming the center of tumor biomarkers. There has been increasing consideration given to targeting the key-role genes in the pathogenesis and treatment of HCC. As a result, novel evidence-based treatments for HCC are immediately required. Altered cell signaling pathways contributing to cell cycle, differentiation, and apoptosis are potential biomarkers of HCC development [35]. Recently, biological studies have pointed to aberrant rat sarcoma virus (RAS)/rapidly accelerated fibrosarcoma protein (RAF)/mitogen-activated and extracellular-signal-regulated kinase (MEK)/extracellular signal-regulated kinase (ERK) signaling pathway activation as being critical for cancer progression, proliferation, and survival, as well as for targeted therapy resistance mechanisms [36]. Many study results revealed that the activation of the Ras/Raf/MEK/ERK pathway may lead to HCC progression functionally. After activation, the pathway promotes transcription of genes involved in tumor proliferation [36, 37]. Viral hepatitis also plays a critical role in the activation of the Ras/Raf/MEK/ERK cascade in HCC. HBV X protein has also been shown to have an important effect on the development of HCC through activating the Ras/Raf/MEK/ERK pathway [38]. Therefore, it is substantial to identify suitable predictive molecular biomarkers in order to customize the early diagnosis and effective treatment of HCC. In the present study, after Venn-Diagram analysis, seven representative common genes were identified: TYMP, TYROBP, CD14, TGFBI, LILRA2, GNLY, and GZMB. Recently, several different biomarkers, including CXCR2, CCR2, EP400, SELENBP1, SLC4A1, SLC26A8, HSPA8P4, CALM1, RPL7p24, FLNA, and CLU have been reported to be associated with the progression of HCC [25, 27, 32]. Nevertheless, no investigation has investigated the expression levels of the seven aforementioned DEGs in PBMC of HBV-HCC patients. After initial screening using RNA-seq, qRT-PCR data then revealed that the expression levels of six DEGs were significantly higher in the patients with HCC compared with those with CHB and healthy controls. Only 1 DEG (CD14) was downregulated. Combined with the ROC analysis results, these data feasibly indicate that novel biomarker and their associated immune responses in HBV-HCC patients may be responsible for pathophysiological progression.

TYMP (thymidine phosphorylase), a nucleoside metabolism enzyme that catalyzes the conversion of thymidine to thymine and 2-deoxyribose-1-phosphate, plays an important role in tumor angiogenesis, apoptosis, and cell proliferation [39]. TYMP is overexpressed in several human cancers [40], and clinical retrospective analysis conducted by Zhang et al. revealed that HCC tumor tissues have higher TYMP expression [41]. Interestingly, our previous network-based study demonstrated that TYMP was upregulated in PBMC of HCC patients. TYMP is mainly recognized as having a strong angiogenic effect [42]. The angiogenic function of TYMP is related to its enzymatic activity. The crosstalk between adaptive immune cells and the cancer endothelium is critical for tumor immune surveillance and the success of immune-based therapies that harness immune cells to kill tumor cells [43]. TYMP stimulates the phosphorylation of focal adhesion kinase and subsequently induces endothelial cell migration through its metabolite 2-D-deoxyribose [44]. TYMP may induce angiogenesis by stimulating the expression of proangiogenic factors, including vascular endothelial growth factor, matrix metalloproteinase, tumor necrosis factor, and interleukin-8 [42]. Overall, this study suggests that TYMP might serve as a potential marker of poor prognosis in HCC.

TYROBP (TYRO protein tyrosine kinase-binding protein) encodes a transmembrane signaling polypeptide and acts as an activating signal transduction element [45]. Previous investigations have indicated that TYROBP is overexpressed and related to tumor progression in several cancers such as glioblastoma, gastric cancer, osteosarcoma, and breast cancer [46–49]. Liu et al. indicated that TYROBP is the critical gene and might be a potential therapeutic target in HCC [50]. The CD14 is expressed by mononuclear cells, macrophages, and dendritic cells as a cell membrane or can be secreted in a soluble form [51]. In our study, consistent with the RNA-seq analysis, qRT-PCR showed downregulation of CD14 with more severe liver disease. Guo et al. showed that CD14 transcripts were significantly lower in the serum and tissue samples of HCC [52], demonstrating CD14 ability to distinguish HCC from liver cirrhosis, so it may be a potential biomarker for CD14 in HCC diagnosis. The TGFBI (transforming growth factor-beta-induced protein), a secreted protein, is induced by TGF-*β* in various human cell types. TGFBI is importantly involved as a regulator of a wide range of biological processes including, cell proliferation, differentiation, inflammation, cell adhesion, and migration [53, 54]. More recently, some cancers are associated with increased TGFBI expression [55]. Based on the Oncomine and Tumor Immune Estimation Resource (TIMER) databases, TGFBI was upregulated in HCC compared to adjacent normal tissues [55]. Our results revealed that TGFBI was highly expressed in PBMC of HCC patients compared with healthy controls. In line with the ROC analysis data, TGFBI may serve as a biomarker for the diagnosis of HCC.

LILRA2 (leukocyte immunoglobulin-like receptor subfamily A member (2)) mediates the regulation of the functions of several types of immune cells and primarily myeloid cells and induces the proinflammatory cytokines [56]. Among the 7 DEGs were two that encode proteins with known functions in the defense and inflammatory mediated responses, including granulysin (GNLY), a defensin-like cytolytic molecule, and granzyme B (GZMB), a serine protease that is associated with GNLY in a variety of cytotoxic mechanisms. Some studies demonstrated a correlation between GNLY and GZMB expressions and clinical outcomes in patients with several cancers [57, 58]. However, the expression of GNLY and GZMB genes in the PBMCs of patients with HCC has not still been reported. It is possible that the dysregulation of these genes may somewhat reflect liver damage. Combined with the ROC analysis results, these genes may serve as biomarkers for the early diagnosis of HCC.

The findings of this study have to be seen in the light of some limitations. One of the most formidable impediments, if not arguably the most, was to obtain large samples from patients, especially for RNA-seq analysis. Financial restrictions along with banking sanctions result in challenging circumstances. In this regard, taking into consideration the fact that the RNA-seq process is costly, a lower number of participants in this study were inevitable. This might have affected the results on the gene expression profiles in all HBV cases and control groups. Alternatively, we utilize the PBMC in the current study, which may be consequent to a wide range of limitations by itself. Of note, the employment of liver samples along with PBMC could lead to precise results. However, the scarcity of liver samples, the expenses for extraction, and jeopardizing the patients' lives for obtaining samples are just a few reasons we did not take advantage of the patient liver sample. Second, findings and conclusions in this study are derived from bioinformatics analysis and then are validated by experimental analysis. However, further external validation is needed. Hence, more validation tests may lead to improvement of credibility of our results. Lastly, dysregulation of gene expression patterns obtained from RNA-seq analysis may be restricted because several genomic alterations eventuate at different levels such as posttranscriptional and posttranslational levels and also metabolic levels. It would be fascinating to incorporate these findings into future researches in order to provide a vigorous molecular perspective of HCC pathogenesis. Biomarkers need to be highly specific for HCC, and the use of several such biomarkers of HCC will be important for an overall diagnosis program that is both sensitive and specific. Sufficient planning of investigations and longitudinal prospective studies are necessary for the future. Biomarker-based screening can be implemented as a one-time event or repeatedly at intervals over time.

These data reveal that a high-throughput gene expression study may tentatively detect the crucial genes implicated in the diagnosis and pathogenesis of HCC. Because these genes were highly dysregulated in the PBMCs of patients with HCC and had a high area under the ROC values, the present study offers that validated genes may serve as a suitable diagnostic biomarker of HCC. However, more evaluations are required to fully understand this finding. Future prospective studies are essential to support the promising association between identified genes and treatment response. Our results proved the accuracy of biomarkers on a small scale; however, large-scale clinical studies are required to statistically validate the feasibility of biomarkers in HCC patients with poor prognosis. Integrated RNA-seq studies provide better understanding and identification of the important mRNAs involved in cancer progression and invasion. Furthermore, RNA-seq enables the identification of promising prognostic and predictive cancer biomarkers for better assessment and follow-up of cancer patients.

Taken together, our gene expression analysis of PBMCs from hepatitis B related HCC patients has demonstrated the presence of alterations, and additional qRT-PCR verification and receiver operating characteristic curve analysis have revealed that seven genes are differentially expressed in the patients with HCC (TYMP, TYROBP, CD14, TGFBI, LILRA2, GNLY, and GZMB). This study may help to understand the progression of hepatitis B diseases progression and provide us with new horizons into the biological function and important prospective clinical diagnosis and prognosis of HCC in the near future.

## Figures and Tables

**Table 1:** PCR primers for quantitative real-time PCR.

Gene name		Primer sequence (5′ to 3′)
TYMP	F	TGGACAAGCATTCCACAGGG
	R	CGCTGATCATTGGCACCTTG
TYROBP	F	GACTGTGGGTGGTCTCAGC
	R	TTCAAGGTTTGGGGGTGCTT
CD14	F	AGCCTAGACCTCAGCCACAA
	R	CTTGGCTGGCAGTCCTTTAG
TGFBI	F	TGCTCCCACAAATGAAGCCT
	R	GCCTCCGCTAACCAGGATTT
LILRA2	F	TGGGGACCTACAGATGCTACA
	R	CTTGTTTTGTGATGGGCTGA
GNLY	F	GTACTACGACCTGGCAAGAGCC
	R	TCAGACAGGTCCTGTAGTCACG
GZMB	F	GGTGGCTTCCTGATACAAGACG
	R	GGTCGGCTCCTGTTCTTTGAT
GAPDH	F	TTCCACCCATGGCAAATTCC
	R	AGGCCATGCCAGTGAGCTTC

**Table 2:** The demographic and clinical properties of the enrolled subjects for RNA-sequencing (*n* = 20).

Variable	All subject (*n* = 20)	Healthy (*n* = 5)	CHB (*n* = 5)	Cirrhosis (*n* = 5)	HCC (*n* = 5)	*P* value
Sex						0.895
Male	11 (55%)	3 (60%)	2 (40%)	3 (60%)	3 (60%)	
Female	9 (45%)	2 (40%)	3 (60%)	2 (40%)	2 (40%)	
Age	48.85 ± 11.88	44.2 ± 6.22	44.4 ± 17.81	51.60 ± 6.34	57.80 ± 5.63	0.157
BMI	27.15 ± 4.91	25.38 ± 4.14	25.62 ± 7.58	31.20 ± 2.77	26.40 ± 2.06	0.202
ALT (U/L)	71.80 ± 48.55	25.40 ± 2.70	27.02 ± 4.84	122.04 ± 14.66	58.80 ± 13.88	<0.001
AST (U/L)	78.50 ± 55.14	26.02 ± 3.53	28.20 ± 6.76	140.40 ± 21.68	68.01 ± 12.02	<0.001
HBeAg + ve	6 (30%)	0 (0%)	0 (0%)	3 (60%)	3 (60%)	0.033
Viral load log_10_ (Copies/mL)	(Median: 55331 Range: 2647–9560679)	—	(Median: 5123 Range: 2647–9691)	(Median: 59618 Range: 13200–102180)	(Median: 5051258 Range: 623776–9560679)	0.02

**Table 3:** The demographic and clinical properties of the enrolled validation cohort (*n* = 100).

Variable	All subject (*n* = 100)	Healthy (*n* = 25)	CHB (*n* = 25)	Cirrhosis (*n* = 25)	HCC (*n* = 25)	*P* value
Sex						0.580
Male	60 (60%)	13 (52%)	15 (60%)	14 (56%)	18 (72%)	
Female	40 (40%)	12 (48%)	10 (40%)	11 [44]	7 (28%)	
Age	49.4 ± 10.86	47.3 ± 6.056	43.5813.80	54.09 ± 8.837	53.3 ± 10.04	0.061
BMI	26.10 ± 4.56	24.66 ± 3.15	27.57 ± 6.51	25.94 ± 3.69	26.25 ± 3.94	0.536
ALT (U/L)	64.82 ± 48.15	21.57 ± 1.51	27.67 ± 5.38	133.78 ± 20.04	65.67 ± 24.32	<0.001
AST (U/L)	70.72 ± 55.10	23.44 ± 3.43	29.33 ± 6.24	152.44 ± 22.67	77.67 ± 26.62	<0.001
HBeAg + ve	30 (30%)	0 (0%)	0 (0%)	14 (56%)	16 (64%)	<0.001
Viral load log_10_ (IU/mL)	(Median: 92750 Range: 5123–9456770)	—	(Median: 9647 Range: 5123–55507)	(Median: 93200 Range: 30679–792054)	(Median: 8278775 Range: 547893–9456770)	<0.001

**Table 4:** Qualitative analysis results of RNA-seq of PBMCs from patients with CHB, cirrhosis, HCC, and healthy controls.

Sample code	Total reads (paired end)	Clean reads (paired end)	Uniquely mapped reads	Uniquely mapped percentage (%)	Gene number
Normal-51	24,058,388	23,995,520	22,455,801	93.58	20,339
Normal-52	21,449,854	21,407,290	19,707,576	92.06	21,281
Normal-53	19,876,972	19,833,714	18,424,909	92.89	18,231
Normal-54	20,780,644	20,739,585	19,357,911	93.33	17,779
Normal-55	21,985,355	21,919,459	20,426,461	93.18	19,127
CHB-45	20,775,086	20,730,564	19,157,914	92.41	19,189
CHB-46	21,055,322	20,988,058	19,601,278	88.63	17,941
CHB-47	20,650,386	20,584,647	19,328,022	93.39	19,336
CHB-48	19,623,356	19,578,662	18,001,907	91.94	18,252
CHB-49	19,430,539	19,379,484	18,027,943	93.02	21,214
Cirrhosis-60	20,628,604	19,561,090	18,038,418	82.21	18,929
Cirrhosis-61	29,921,566	29,845,514	28,031,232	93.92	20,486
Cirrhosis-62	22,369,942	22,302,971	20,784,668	93.19	19,787
Cirrhosis-63	23,396,442	23,314,384	21,668,246	92.93	18,411
Cirrhosis-64	25,556,948	25,462,716	23,864,212	93.72	17,776
HCC-72	22,118,385	22,044,356	20,535,946	93.15	22,472
HCC-73	23,978,566	23,8141,72	22,213,130	93.27	19,428
HCC-74	25,877,964	25,793,604	24,152,401	93.63	21,556
HCC-75	29,793,379	29,695,525	27,988,801	94.25	20,973
HCC-76	23,349,876	23,300,209	21,870,234	93.86	23,387

**Table 5:** Simple topological parameters of PPIs obtained from Cytoscape software.

Parameter	CHB-cirrhosis	Normal-cirrhosis	Normal-HCC	CHB-HCC
Number of nodes	408	1262	1450	772
Number of edges	149	2100	1816	410
Network diameter	5	5	6	6
Network radius	3	3	3	3
Network centralization	0.154	0.189	0.214	0.133
Shortest paths	12656 (7%)	653672 (40%)	704760 (33%)	72630 (12%)
Characteristics path length	2.821	2.744	2.821	2.920
Network density	0.002	0.000	0.002	0.001
Clustering coefficient	0.023	0.137	0.114	0.036
Network heterogeneity	5.525	5.0.35	5.920	5.962
Number of connected components	296	456	613	503
Average number of neighbors	0.676	3.285	2.460	1.023

**Table 6:** The top hub genes for each constructed PPIs.

CHB cirrhosis				Normal cirrhosis			
Gene	Degree	BC*∗*	CC*∗*	Gene	Degree	BC	CC
E2F1	422	0.7057846	0.60215054	CEBPB	72	0.21700292	0.50217526
TAL1	313	0.35365633	0.5258216	ELF1	28	0.18949992	0.49029126
ETS1	304	0.21712687	0.4057971	E2F1	22	0.17340145	0.4750147
MYBL2	282	0.11567915	0.38487973	MYC	21	0.17279657	0.49208283
IRF4	262	0.07313807	0.34890966	SPI1	5	0.17593123	0.48296473
NFE2	229	0.06093951	0.39857651	USF1	3	0.16068855	0.51728553
HAGH	207	0.03797363	0.47659574	CHD2	3	0.09980585	0.45675523
TANGO2	165	0.02242236	0.46861925	TCF7L2	3	0.07070757	0.43324397
SMIM5	150	0.02242236	0.46861925	POU2F2	3	0.0589139	0.44371225
AIF1	147	0.05272269	0.39575972	TAL1	3	0.05890397	0.45013928

Normal-HCC				CHB-HCC			
Gene	Degree	BC	CC	Gene	Degree	BC	CC

ELF1	313	0.324255	0.51314985	USF1	103	0.47779082	0.53585657
RAD21	267	0.30769676	0.4964497	CHD2	96	0.43900845	0.538
USF1	212	0.21050533	0.49940476	NFIC	54	0.20378267	0.45439189
CHD2	173	0.18058038	0.50756201	ZBTB7A	52	0.166987	0.38985507
E2F4	130	0.11268231	0.45722071	ETS1	46	0.13426444	0.37569832
NFIC	100	0.07560456	0.45107527	STAT1	33	0.14143326	0.43954248
ZBTB7A	95	0.05740561	0.36541812	JUNB	12	0.03882081	0.42097027
ETS1	90	0.04703516	0.38663594	CIRBP	5	0.01451378	0.46701389
JUN	70	0.05130947	0.41929035	SMAD3	5	0.01943931	0.42163009
ELK1	65	0.04976925	0.44818376	ZFP36	5	0.01269741	0.4317817

*∗*BC: betweenness centrality; CC: closeness centrality.

**Table 7:** List of the seven common genes identified by Venn-Diagram analysis.

DEGs	Description	Log_2_ fold change (log_2_FC)		
		CHB vs. healthy	Cirrhosis vs. healthy	HCC vs. healthy
TYMP	Thymidine phosphorylase	4.12	4.60	6.63
TYROBP	TYRO protein tyrosine kinase-binding protein	3.94	4.96	6.12
CD14	Cluster of differentiation 14	−3.88	−4.23	−5.70
TGFBI	Transforming growth factor beta induced	2.55	3.93	5.11
LILRA2	Leukocyte immunoglobulin-like receptor subfamily A member 2	3.91	4.10	4.52
GNLY	Granulysin	3.06	4.33	4.86
GZMB	Granzyme B	2.86	4.02	4.33

## Data Availability

The data can be acquired from the corresponding author.

## Additional Points

*Study Highlights*. We investigated generating profiles throughout using the expression of PBMC using the RNA-sequencing method and compared the gene expression between HCC patients and other HBV-related diseases and healthy controls. Results indicated that the differentially expressed genes that were implicated in the immune responses and shared by HCC, liver cirrhosis, and CHB could be more appropriate for the diagnosis of the HCC. The expression of seven selected genes (TYMP, TYROBP, CD14, TGFBI, LILRA2, GNLY, and GZMB) were validated by qRT-PCR, and diagnostic accuracy for HCC was obtained by ROC analysis.
